# Letter to the Editor

**Published:** 1984-01

**Authors:** J. S. Hughes Games

**Affiliations:** Clifton


### Letter to the Editor

Sir, 1st December, 1983

I was fascinated in reading through your journal of July. In the extract from '100 years ago' quoting my grandfather, George Munro Smith and his observations about the cardiograph in medicine and saying 'so little has been done especially in England in what might become a fruitful field of clinical work'.

He was a man of tremendous originality and enthusiasm and it fascinates me that he, as a surgeon, should have appreciated the possibilities of cardiographic tracings before many of his colleagues.

Yours faithfully,
Dr. J. S. Hughes Games
Clifton

P.S. His daughter, a very lively minded 87-year-old, lives in Congresbury.

